# The lysophosphatidic acid receptor LPA_4_ regulates hematopoiesis-supporting activity of bone marrow stromal cells

**DOI:** 10.1038/srep11410

**Published:** 2015-06-19

**Authors:** Hidemitsu Igarashi, Noriyuki Akahoshi, Takayo Ohto-Nakanishi, Daisuke Yasuda, Satoshi Ishii

**Affiliations:** 1 Department of Immunology, Akita University Graduate School of Medicine, Akita, Japan

## Abstract

Lysophosphatidic acid (LPA) is a pleiotropic lipid mediator that acts through G protein-coupled receptors (LPA_1-6_). Although several biological roles of LPA_4_ are becoming apparent, its role in hematopoiesis has remained unknown. Here, we show a novel regulatory role for LPA_4_ in hematopoiesis. *Lpar4* mRNA was predominantly expressed in mouse bone marrow (BM) PDGFRα^+^ stromal cells, known as the components of the hematopoietic stem/progenitor cell (HSPC) niche. Compared with wild-type mice, LPA_4_-deficient mice had reduced HSPC numbers in the BM and spleen and were hypersusceptible to myelosuppression, most likely due to impairments in HSPC recovery and stem cell factor production in the BM. Analysis of reciprocal BM chimeras (LPA_4_-deficient BM into wild-type recipients and *vice versa*) indicated that stromal cells likely account for these phenotypes. Consistently, LPA_4_-deficient BM stromal cells showed downregulated mRNA expression of stem cell factor and tenascin-c *in vitro*. Taken together, these results suggest a critical and novel role for the LPA/LPA_4_ axis in regulating BM stromal cells.

Lysophosphatidic acid (LPA) is a bioactive lysophospholipid that is composed of a phosphate, a glycerol and a fatty acid^1^. LPA has roles in many cellular functions, including proliferation, migration, cytokine secretion and morphological changes^2^,^3^. LPA is known to be a major growth factor in serum^4^. Under physiological conditions, LPA in human plasma reaches approximately 80–100 nM^5^. A primary mechanism of LPA production involves lysophospholipase D activity of the plasma enzyme autotaxin, which converts lysophosphatidylcholine and other lysophospholipids to LPA^6^,^7^. To date, at least 6 subtypes of LPA-specific receptors have been identified^8^, and they are divided into two families on the basis of their amino acid sequence homology. LPA_1_/Edg-2/Vzg-1^9^, LPA_2_/Edg-4^10^ and LPA_3_/Edg-7^11^ are members of the Edg family of LPA receptors, whereas LPA_4_/GPR23/p2y9^12^, LPA_5_/GPR92^13^ and LPA_6_/p2y5^14^ form a distinct family of LPA receptors, *i.e*., the non-Edg family of LPA receptors^15^,^16^. All LPA receptors are G protein-coupled. Each receptor couples to multiple, but specific, G proteins, including G12/13, Gi/o and Gs, that modulate a variety of intracellular signaling molecules^15^,^17^.

Following the identification of the orphan receptor GPR23/p2y9 as LPA_4_^12^, our research group found that LPA_4_ deficiency in mice results in a partial embryonic lethal phenotype due to the abnormal development of blood and lymphatic vessels^18^. Additionally, an independent research group reported that LPA_4_-deficient mice have increased trabecular bone density compared to wild-type (WT) mice, suggesting that LPA_4_ has an inhibitory effect on the osteogenic differentiation of mesenchymal stem cells (MSCs)^19^. MSCs are multipotent and can differentiate not only into mesenchymal lineages, including osteoblasts, adipocytes and chondrocytes, but also into endothelium, neurons and skeletal muscle^20^. Indeed, human MSCs have been shown to express *Lpar4* mRNA^19^. In addition, the BioGPS gene expression database (http://www.biogps.org) indicates that the mouse *Lpar4* gene is expressed in mesenchymal cells such as osteoblasts and C3H10T1/2 cells. Thus, LPA_4_ is presumed to play pivotal roles in various cellular processes of mesenchymal cells in multiple tissues. However, this has not yet been thoroughly investigated.

The bone marrow (BM) is the main hematopoietic organ in adult mammals. In the BM, the hematopoietic stem/progenitor cells (HSPCs) give rise to all blood cell lineages. The maintenance, differentiation and proliferation of HSPCs are regulated in both cell-autonomous and non-cell-autonomous fashions^21^. The non-cell-autonomous regulation of HSPCs requires factors important for the proliferation, mobilization, homing and engraftment of hematopoietic stem cells (HSCs); such factors are produced by various cells surrounding HSCs^22^. This local microenvironment is called the HSPC niche. The HSPC niche is subdivided into two types, the osteoblastic and perivascular niches^22^. The cellular components of the perivascular niche have been reported to be mesenchymal stromal cells, such as CXCL12-abundant reticular cells (CAR cells)^23^ and nestin^+^ MSCs^24^. Although they are essential for HSPC maintenance, the molecules regulating mesenchymal stromal cells are not yet fully understood^25^.

In this report, we observed that LPA_4_-deficient mice were highly sensitive to myelosuppression and showed a delay in the recovery of HSPC numbers. LPA_4_ was predominantly expressed in the BM mesenchymal stromal cells. In addition, LPA_4_ in the BM mesenchymal stromal cells was shown to regulate the production of factors involved in HSPC proliferation both *in vivo* and *in vitro*. Our present study consistently demonstrates a significant role for LPA_4_ in maintaining the HSPC niche.

## Methods

### Mice

LPA_4_-deficient mice on a C57BL/6 genetic background have been described previously^18^. C57BL/6 mice congenic for the Ly5 locus (B6-Ly5.1) were purchased from Sankyo Labo Service (Tokyo, Japan) by permission of Prof. Hiromitsu Nakauchi (Institute of Medical Science, The University of Tokyo). Male mice of 9–12 weeks old were used for these studies. Mice were housed under specific pathogen-free conditions in an air-conditioned room and fed standard laboratory chow *ad libitum* (CE-2; CLEA Japan, Tokyo, Japan), in accordance with institutional guidelines. All experimental procedures were approved by the Institutional Animal Care and Use Committee of Akita University.

### Myelosuppression models

Mice were intravenously injected with 5-fluorouracil (5-FU; Kyowa Hakko, Tokyo, Japan) at a dose of 250 mg/kg body weight, and their survival was monitored for 24 days. Some mice were euthanized for analysis of the BM cells and peripheral blood (PB) on days 0, 2, 4, 7 and 10. For experiments with the BM chimeric mice (shown below), mice were intraperitoneally injected with 5-FU (150 mg/kg body weight) twice, at a 1-week interval, and their survival was monitored for 24 days after initial injection. In another model, mice were irradiated with 8 Gy in two split doses with a 3-hr interval using a cabinet X-ray system CP-160 (Faxitron X-ray Corporation, Wheeling, IL). Their survival was then monitored for 24 days.

### PB collection

Under isoflurane anesthesia, PB was collected from the retro-orbital sinus using capillaries and analyzed using a hematological analyzer (Celltac MEK-5258, Nihon Kohden, Tokyo, Japan).

### Stromal and hematopoietic cell isolation

Hematopoietic BM cells were obtained from femurs and tibias by flushing the bones with PBS containing 0.5% BSA (Sigma-Aldrich). For isolation of stromal cells, the flushed bones were minced with scissors. Then, the bone fragments were incubated with DMEM (Sigma-Aldrich) containing 10% FBS (Gibco) and 3 mg/ml type I collagenase (Worthington Biochemical, Lakewood, NJ) for 60 min at 37 °C. The cell suspensions were filtered with a 100-μm cell strainer. Red blood cells were lysed using BD Pharm Lyse Buffer (BD Biosciences).

### Flow cytometry and cell cycle analysis

The cell suspension was preincubated with Fc block (BD Biosciences) to avoid nonspecific binding of antibodies. The following primary antibodies were used: anti-CD3 (145-2C11), anti-CD4 (RM4-5), anti-CD8 (53-6.7), anti-CD11b (M1/70), anti-B220 (RA3-6B2), anti-Gr-1 (RB6-8C5), anti-Ter119 (TER-119), anti-CD45 (30-F11), anti-FcRγII-III (93), anti-Sca-1 (E13-161.7), anti-c-Kit (2B8), anti-CD41 (MWReg30), anti-CD48 (HM-48-1), anti-CD150 (TC15-12F12.2), anti-IL7Rα (A7R34), anti-Flt3 (A2F10), anti-PDGFRα (APA5) (all from BioLegend, San Diego, CA) and anti-CD34 (RAM34; eBioscience, San Diego, CA). A mixture of CD4, CD8, CD11b, B220, Ter-119 and Gr-1 antibodies was used as the lineage (Lin) mixture. 7-AAD was used to identify and exclude dead cells. For cell cycle analysis, the cells were fixed and permeabilized using the Fixation/Permeabilization Solution kit (BD Biosciences) and were stained with PE-conjugated anti-Ki-67 antibody (BD Biosciences) and DAPI (BioLegend). The stained cells were analyzed and sorted using a FACSAria and an Accuri C6 flow cytometer (BD Biosciences), respectively. The flow cytometry data were analyzed using FlowJo ver. 10.0.5 (Treestar, Ashland, OH).

### Colony-forming units assay

The nucleated 2 × 10^4^ BM cells were plated in methylcellulose media supplemented with a cocktail of recombinant cytokines (Methocult 3434; StemCell Technologies, Vancouver, Canada). Cultures were plated in duplicate and placed in a humidified chamber with 5% CO_2_ at 37 °C. Colonies containing at least 30 cells were counted at day 12 of culture.

### Bone marrow transplantation

Lethally irradiated mice were transplanted with 2 × 10^6^ BM cells. Three months later, their peripheral blood was collected from retro-orbital venous plexus. The percentages of donor-derived cells were analyzed with an Accuri C6 flow cytometer (BD Biosciences) using anti-CD45.1 (A20) and anti-CD45.2 (104) antibodies (BioLegend), detecting Ly5.1 and Ly5.2, respectively.

### Cell culture

The BM stromal cells isolated by collagenase treatment were maintained in α-MEM GlutaMax (Gibco) containing 10% FBS, 10% horse serum (Gibco) and 100 U/ml penicillin/streptomycin (Wako Chemicals, Osaka, Japan) for 7 days. The cells were starved in α-MEM GlutaMax containing 0.1% BSA, 10 μM HA130 (an inhibitor of autotaxin; Calbiochem) and 100 U/ml penicillin/streptomycin for 12 hr to exclude hematopoietic cells. The starved cells were stimulated with 1-oleoyl LPA (Avanti Polar Lipids, Alabaster, AL) at a final concentration of 10 μM by adding an equivalent volume of medium containing 20 μM 1-oleoyl LPA and incubated for 12 hr.

### Quantitative reverse transcription-PCR

For preparation of cDNA templates from the cultured cells and sorted cells, total RNA was isolated with the RNAqueous-micro kit (Ambion, Austin, TX) or the RNeasy mini kit (Qiagen, Valencia, CA) and subjected to oligo-dT- and random hexamer-primed reverse transcription with the Primescript enzyme (Takara Bio, Otsu, Japan). Quantitative PCR was performed using a LightCycler 480 instrument (Roche Diagnostics) with the SYBR Premix ExTaq II (Takara Bio) and KAPA SYBR Fast qPCR Kit (Kapa Biosystems, Wilmington, MA). The mRNA levels were normalized to *Hprt1* or *Rn18s* as a standard housekeeping gene. The primer sequences are listed in Supplementary Table S1. The PCR program was as follows: denaturation at 95 °C for 30 sec and 50 cycles of amplification consisting of denaturation at 95 °C for 10 sec and annealing and extension at 60 °C for 20 sec.

### ELISA

At days 0 and 9 after 5-FU administration, the femurs were flushed with 500 μl of ice-cold PBS or 300 μl of ice-cold PBS containing 1% NP40 and a protease inhibitor cocktail to measure the protein levels of CXCL12 or SCF, respectively. The fluids were centrifuged at 500 × *g* for 5 min. Then, the supernatant was subjected to ELISAs for CXCL12 and SCF using the DuoSet ELISA kits (R&D systems) according to the manufacturer’s instructions.

### Statistical analysis

Data are expressed as the mean ± SEM and analyzed using GraphPad Prism 6 software (GraphPad Software). All data were combined from two or three independent experiments. The two-tailed unpaired Welch’s *t*-test, log-rank test or two-way ANOVA followed by Bonferroni’s post-hoc test was used for comparisons between 2 groups. One-way ANOVA followed by Tukey’s post-hoc test was used for comparisons among 3 groups. Values of *P* < 0.05 were considered statistically significant.

## Results

### In mouse BM, LPA4 is predominantly expressed in PDGFRα^+^ stromal cells

**Figure 1:**
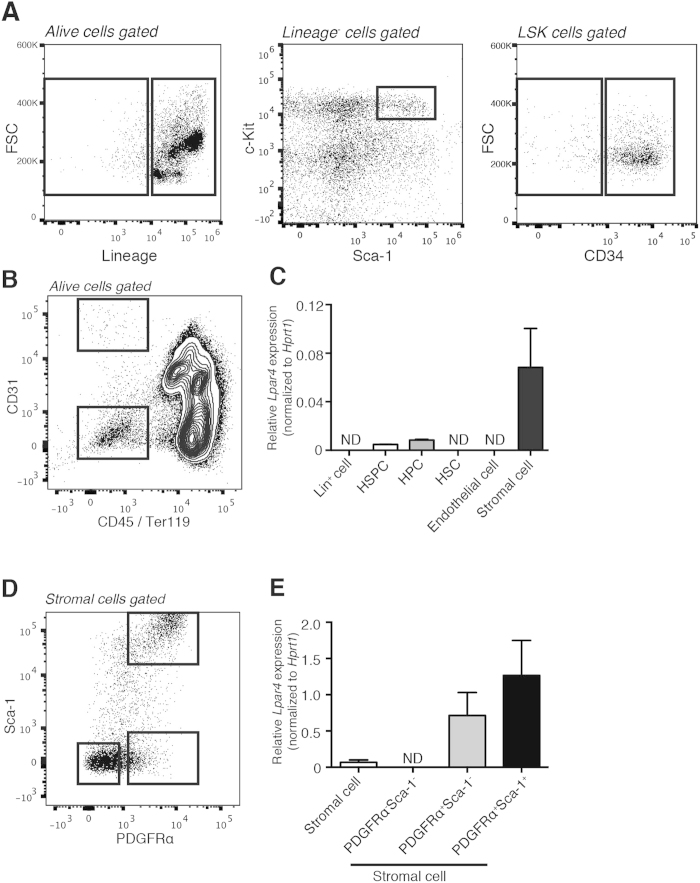
*LPA_4_* expression in the BM. (**A**) Representative flow cytometry gating of Lin^+^ mature hematopoietic cell, Lin^−^Sca-1^+^c-Kit^+^ HSPC, CD34^+^Lin^−^Sca-1^+^c-Kit^+^ HPC and CD34^−^Lin^−^Sca-1^+^c-Kit^+^ HSC populations. The BM cells were obtained by flushing tibias and femurs and were analyzed by flow cytometry. (**B**) Representative flow cytometry gating of CD45^−^Ter119^−^CD31^+^ endothelial cell and CD45^−^Ter119^−^CD31^−^ stromal cell populations. The BM cells were obtained from femurs and tibias by collagenase treatment and were analyzed by flow cytometry. (**C**) Comparison of *Lpar4* mRNA expression in Lin^+^ cells, HSPCs, HPCs, HSCs, endothelial cells and stromal cells by quantitative RT-PCR analysis (*n* = 6). ND: not detected. (**D**) Three stromal cell subsets based on PDGFRα and Sca-1 expression. A representative FACS plot shows PDGFRα^−^Sca-1^−^, PDGFRα^+^Sca-1^−^ and PDGFRα^+^Sca-1^+^ cell populations. (**E**) Comparison of *Lpar4* mRNA expression in PDGFRα^−^Sca-1^−^, PDGFRα^+^Sca-1^−^ and PDGFRα^+^Sca-1^+^ cells by quantitative RT-PCR analysis (*n* = 6). ND: not detected.

First, we examined the expression level of *Lpar4* mRNA in various populations of mouse BM cells. Lin^+^ mature hematopoietic cells, Lin^−^Sca-1^+^c-Kit^+^ (LSK) HSPCs, CD34^+^ LSK hematopoietic progenitor cells (HPCs) and CD34^−^ LSK HSCs were sorted from the BM cells harvested by flushing tibias and femurs (Fig. 1A). In addition, CD45^−^Ter119^−^CD31^+^ endothelial and CD45^−^Ter119^−^CD31^−^ stromal cells were sorted from the BM cells obtained by collagenase treatment of the flushed bones (Fig. 1B). Hematopoietic cells, including Lin^+^ cells and HSCs, and endothelial cells expressed little or no detectable *Lpar4* mRNA, whereas HSPCs and HPCs expressed low levels of *Lpar4*. Notably, the *Lpar4* mRNA expression level was quite high in the stromal cells (Fig. 1C). Based on the PDGFRα and Sca-1 expression levels, the stromal cells can be divided into three subsets: PDGFRα^−^Sca-1^−^, PDGFRα^+^Sca-1^−^ and PDGFRα^+^Sca^−^1^+^ stromal cells (Fig. 1D). It has been reported that the PDGFRα^+^Sca-1^−^ and PDGFRα^+^Sca-1^+^ subsets include CAR cells and MSCs, respectively^23^,^26^. Furthermore, the PDGFRα^−^Sca-1^−^ subset was reported to consist mainly of osteoblasts^26^. Interestingly, PDGFRα^+^Sca-1^−^ and PDGFRα^+^Sca-1^+^ stromal cells expressed 10–to 15-fold higher levels of *Lpar4* mRNA than the parental unfractionated (CD45^−^Ter119^−^CD31^−^) stromal cells (Fig. 1E). These results demonstrate that the cells expressing *Lpar4* mRNA in the mouse BM are predominantly PDGFRα^+^ stromal cells.

We also examined mRNA expression levels of other LPA receptors in HSPCs and the three subsets of stromal cells. In HSPCs, *Lpar4* mRNA expression level was the lowest among six receptors (Supplementary Fig. S1A). About the stromal cells, *Lpar1* and *Lpar6* mRNA were ubiquitously expressed in all subsets, while *Lpar2*, *Lpar3* and *Lpar5* mRNA were undetectable under our experimental conditions (Supplementary Fig. S1B-D).

### LPA_4_-deficient mice have decreased HSPC number in the BM and spleen

**Figure 2:**
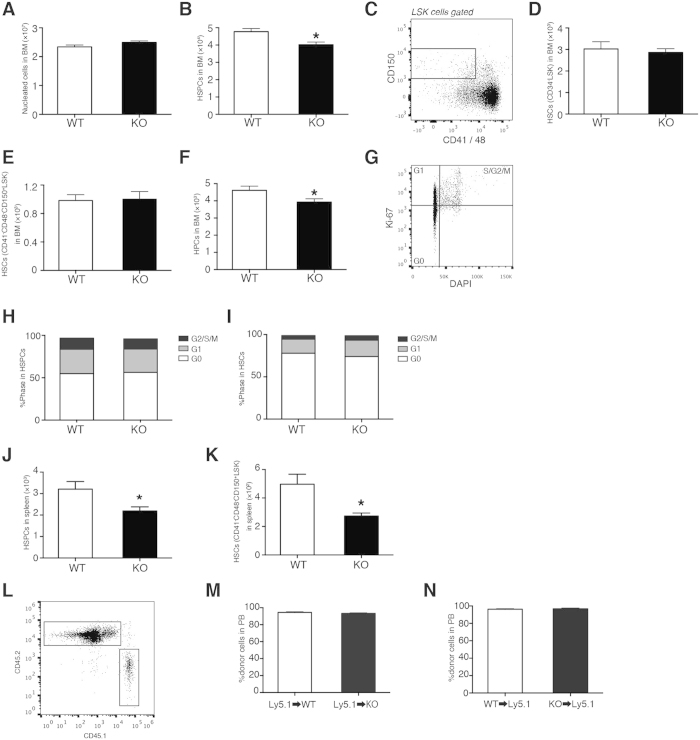
Normal steady-state hematopoiesis in LPA4-deficient mice. (**A-F**) Measurement of hematopoietic parameters in the BM. Total BM cellularity (**A**), HSPC number (**B**), HSC numbers defined as CD34^−^ LSK and CD41^−^CD48^−^CD150^+^ LSK cells (**D**,**E**, respectively) and HPC number (**F**) in the BM (*n* = 9–11). Representative flow cytometry gating of CD41^−^CD48^−^CD150^+^ LSK HSC population is shown in (**C**). (**G**) Cell cycle status assessed through Ki-67 and DAPI staining. (**H,I**) Cell cycle status of HSPCs (**H**) and HSCs (**I**) in the BM (*n* = 9). (**J** and **K**) HSPC number (**J**) and HSC number (**K**) in the spleen (*n* = 8–9). (**L**) FACS analyses of donor-derived cells in PB three months after BM transplantation. (**M**) Analyses of BM chimeras of WT and LPA_4_-deficient mice reconstituted with Ly5.1 WT BM (*n* = 8–9). (**N**) Analyses of BM chimeras of Ly5.1 WT mice reconstituted with WT or LPA_4_-deficient BM (*n* = 7–8). *P < 0.05.

Because the stromal cells form an important constituent of the perivascular niche, we analyzed hematopoietic parameters of LPA_4_-deficient mice under homeostatic conditions, including total BM cellularity and numbers of hematopoietic stem/progenitor cells and mature cells. The total BM cellularity was normal in LPA_4_-deficient mice (Fig. 2A). The numbers of CD3^+^CD4^+^, CD3^+^CD8^+^ and B220^+^ lymphocytes in the BM were also unchanged, but the number of CD11b^+^Gr-1^+^ granulocytes/monocytes was significantly higher in the BM of LPA_4_-deficient mice than in that of WT mice (Supplementary Fig. S2A-G). We further observed that the number of HSPCs was significantly lower in LPA_4_-deficient mice than in WT mice (Fig. 2B). However, the number of HSCs, which are defined as CD34^−^ LSK (Fig. 1A) or CD41^−^CD48^−^CD150^+^ LSK cells (Fig. 2C), was normal in LPA_4_-deficient mice (Fig. 2D and E, respectively). Therefore, we reasoned that the HPC number is decreased in these mice (Fig. 2F). Next, we examined whether the decrease in HPC number was due to impaired cell cycle progression of HSCs and HSPCs. However, their cell cycle status in LPA_4_-deficient mice was normal (Fig. 2G-I).

To further characterize HSPCs of LPA_4_-deficient mice, we assessed the hematopoietic colony-forming capacity of the BM cells cultured with cytokines. The colony numbers of LPA_4_-deficient mice were slightly lower than those of WT mice (Supplementary Fig. S3). However, considering the intrinsically smaller number of HSPCs in the BM of LPA_4_-deficient mice (Fig. 2B), the colony-forming capacity seemed to be comparable between WT and LPA_4_-deficient mice. In addition, the expression levels of various transcripts in HSPCs were measured. Among the genes examined, *Cdk2* and *Spi1* (encoding PU.1) were down-regulated significantly in HSPCs of LPA_4_-deficient mice compared with those of WT mice (Supplementary Fig. S4).

We next examined the numbers of Lin^−^IL7Rα^+^Flt3^+^ common lymphoid progenitors (Supplementary Fig. S2H), Lin^−^Sca-1^−^c-Kit^+^CD34^+^FcRγII-III^high^ granulocyte and macrophage progenitors and Lin^−^Sca-1^−^c-Kit^+^CD34^−^FcRγII-III^low^ megakaryocyte and erythrocyte progenitors (Supplementary Fig. S2I), all of which are differentiated from HPCs. These cell numbers were comparable between the two genotypes (Supplementary Fig. S2J). However, the ratio of granulocyte and macrophage progenitors to HSPCs was significantly increased in LPA_4_-deficient mice (Supplementary Fig. S2K). In mice, adult hematopoiesis occurs not only in the BM but also in the spleen^27^. By analyzing the spleen, we found that LPA_4_-deficient mice have significantly decreased numbers of both HSCs and HSPCs (Fig. 2J-K).

To investigate the contribution of LPA_4_ to BM reconstitution, WT and LPA_4_-deficient mice were lethally irradiated and transplanted with WT or LPA_4_-deficient BM. Three months after transplantation, the chimerism of the recipient mice was determined in PB cells (Fig. 2L). The results showed no significant difference in the percentages of donor cells among these BM chimeras (Fig. 2M-N). Together, these results suggest that LPA_4_ regulates the homeostasis of HSPCs in the BM and spleen.

### LPA_4_-deficient mice show a delay in hematopoietic recovery after myelosuppression

**Figure 3:**
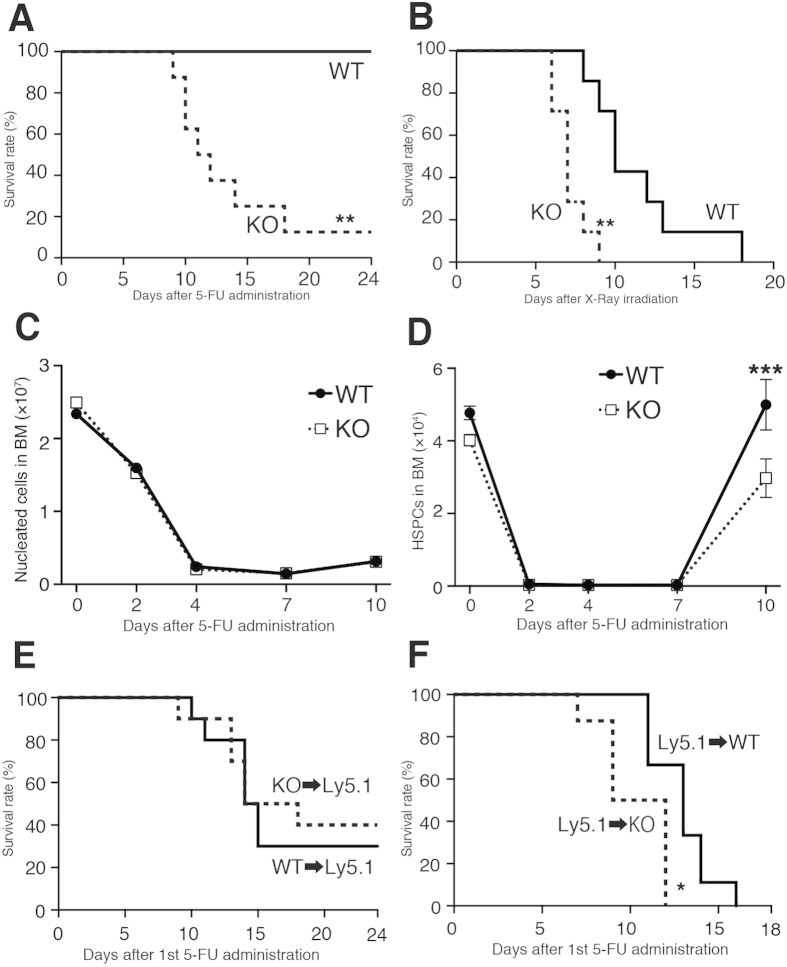
Impaired hematopoietic recovery of LPA_4_-deficient mice after 5-FU administration. (**A**) Survival curves of LPA_4_-deficient and WT mice after 5-FU administration (*n* = 8). (**B**) Survival curves of LPA_4_-deficient and WT mice after X-ray irradiation (*n* = 7). (**C,D**) Kinetics of total BM cellularity (**C**) and HSPC number (**D**) in the BM after 5-FU administration (*n* = 6–11). (**E,F**) Survival curves of BM chimeric mice after 5-FU administration. (**E**) Analyses of Ly5.1 WT mice reconstituted with WT or LPA_4_-deficient BM (*n* = 9). (**F**) Analyses of WT and LPA_4_-deficient mice reconstituted with Ly5.1 WT BM (*n* = 8-9). *P < 0.05. **P < 0.01. ***P < 0.001.

To investigate the function of LPA_4_ under myelosuppression, mice were injected with 5-FU or were sublethally irradiated. LPA_4_-deficient mice displayed significantly higher lethality than WT mice in both myelosuppression models (Fig. 3A-B). After 5-FU administration, the numbers of red blood cells and platelets in PB were reduced similarly in both genotypes (Supplementary Fig. S5A-B). However, the numbers of white blood cells in LPA_4_-deficient mice were reduced earlier than those in WT mice (Supplementary Fig. S5C). At day 10 after 5-FU administration, LPA_4_-deficient mice had a significantly reduced HSPC number in the BM, although the total BM cellularity was unaffected by the drug (Fig. 3C-D).

As described above, HPCs and stromal cells express *Lpar4* mRNA in the BM (Fig. 1C). To determine which cell type is responsible for the hypersusceptibility of LPA_4_-deficient mice to 5-FU, we used the BM chimeric mice shown in Fig. 2M,N. When WT mice transplanted with WT or LPA_4_-deficient BM cells were treated with 5-FU, the lethality was indistinguishable between these two classes of chimeric mice (Fig. 3E). In contrast, when we administered 5-FU to WT and LPA_4_-deficient mice that had undergone transplantation of BM from WT donors, the LPA_4_-deficient recipients were significantly more susceptible to 5-FU than WT recipients (Fig. 3F), recapitulating the results observed with naïve mice. Histological observations of the lung, liver, colon and small intestine 7 days after 5-FU administration revealed that little or no injury occurred in these organs of WT and LPA_4_-deficient mice (data not shown). Thus, these results suggest that LPA_4_ expressed in stromal cells is involved in hematopoietic recovery after myelosuppression.

### LPA_4_ deficiency in BM stromal cells impairs the production of HSPC proliferation factors

**Figure 4:**
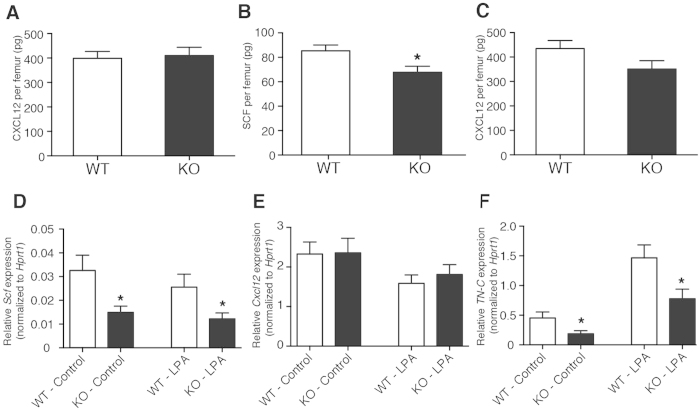
Reduced production of SCF and TN-C in LPA_4_-deficient stromal cells. (**A**) The amount of CXCL12 per femur from naïve LPA_4_-deficient and WT mice (*n* = 9). (**B,C**) The amounts of SCF (**B**) and CXCL12 (**C**) per femur from LPA_4_-deficient and WT mice (*n* = 13–14) at day 9 after 5-FU administration. (**D-F**) Quantitative RT-PCR for *Scf* (**D**) *Cxcl12* (**E**), and *TN-C* (**F**) mRNA expression in isolated BM stromal cells from LPA_4_-deficient and WT mice that were treated with or without LPA (*n* = 9–10). *P < 0.05 vs. WT.

CXCL12 and SCF, important cytokines for the HSPC maintenance and proliferation, are produced predominantly by BM stromal cells^28^,^29^. Because LPA_4_-deficient mice displayed reduced numbers of HSPCs and impaired extramedullary hematopoiesis, we evaluated the CXCL12 and SCF protein levels in the BM of LPA_4_-deficient mice. Under homeostatic conditions, there was no significant difference in the protein levels of CXCL12 between LPA_4_-deficient and WT mice (Fig. 4A). The protein level of SCF was too low to be detected. At day 9 after 5-FU administration, LPA_4_-deficient mice had a significantly lower level of SCF protein in the BM than did WT mice (Fig. 4B), and the production of CXCL12 protein tended to be impaired in LPA_4_-deficient mice (Fig. 4C). These *in vivo* data suggest that LPA_4_ in the stromal cells regulates the SCF protein expression under myelosuppression. Consistent results were obtained with cultures of primary BM stromal cells. The LPA_4_-deficient stromal cells expressed a significantly lower level of *Scf* mRNA than did WT cells (Fig. 4D). In addition, the LPA_4_-deficient stromal cells also showed a significant reduction in the mRNA level of *tenascin-c* (*TN-C*) (Fig. 4F), an extracellular matrix protein that promotes HSPC proliferation^30^. When we stimulated stromal cells with LPA, upregulation of the *TN-C* mRNA level was observed in both WT and LPA_4_-deficient cells (Fig. 4F). In contrast, the expression levels of *Scf* and *Cxcl12* mRNA were unaffected by LPA stimulation (Fig. 4D-E). Together, these results suggest that LPA_4_ in stromal cells regulates the production of proliferation-promoting factors for HSPCs.

## Discussion

In this study, we found that LPA_4_-deficient mice showed hypersusceptibility to myelosuppressive stresses, likely due to impaired stress recovery of the HSPC number in the BM. This impairment was associated with reduced production of SCF in the BM of LPA_4_-deficient mice. Bone marrow chimeric mice showed that the target cells of LPA_4_ signaling were of non-hematopoietic origin. Therefore, it was consistent that the LPA_4_ deficiency in the BM stromal cell cultures suppressed the expression levels of *Scf* and also *TN-C*. SCF potently regulates HSPC proliferation and differentiation through the receptor c-Kit^31^. Previously, PDGFRα^+^Sca-1^−^ cells were shown to produce SCF in the BM and contribute to the formation of the perivascular niche^23^. TN-C is an extracellular matrix protein that is predominantly produced by PDGFRα^+^ stromal cells in the BM and regulates HSPC proliferation through integrin α9^30^. The expression of TN-C was reportedly upregulated after myelosuppressive stress^30^. Similarly to LPA_4_-deficient mice, TN-C-deficient mice were hypersensitive to lethal myelosuppression and showed a delay of hematopoietic recovery^30^. Thus, we assume that the lethality to myelosuppressive stress was dependent, at least in part, on LPA_4_ from the stromal cells. Because *Lpar4* mRNA was predominantly expressed in PDGFRα^+^Sca-1^+^/ PDGFRα^+^Sca-1^−^ stromal cells in the BM, LPA_4_ may regulate the function of PDGFRα^+^ cells by affecting the production of HSPC proliferation factors, although the underlying molecular mechanisms remain to be elucidated.

To date, BM mesenchymal stromal cells have been reported to express at least two G protein-coupled receptors (GPCRs), parathyroid hormone/parathyroid hormone-related peptide receptor (PTH_1_)^32^ and prostaglandin E receptor 4 (EP_4_)^33^, that support HSPC proliferation. Activation of PTH_1_ resulted in the upregulation of the protein expression of Jag1 in α1(I) collagen-expressing osteoblastic cells and then induced HSPC proliferation through Notch1. Meanwhile, EP_4_ activation enhanced the mRNA expression of various mitogenic protein genes, as well as *Jagged1*, in ALCAM^–^Sca-1^+^ mesenchymal progenitor cells. It is interesting to note that both GPCRs are coupled to Gs protein for these phenotypes^25^. However, we observed that LPA_4_ is coupled to G12/13 protein not but to Gs, Gi/o or Gq protein in mouse C3H10T1/2 mesenchymal cells (Keisuke Yanagida and S.I., manuscript in preparation). Consistently, LPA_4_-deficient stromal cells showed no change in *Jagged1* expression level (Supplementary Fig. S6). Thus, our results suggest that LPA_4_ controls the HSPC niche via a novel GPCR signaling pathway in BM stromal cells. Because *Lpar1* and *Lpar6* mRNA were also expressed in PDGFRα^+^ stromal cells in the BM, LPA_1_, LPA_4_ and LPA_6_ may exert redundant functions in the hematopoiesis-supporting activity. Indeed, these three LPA receptors were reported to couple to G12/13 protein^15^.

In steady-state hematopoiesis, LPA_4_-deficient mice showed mildly reduced numbers of HPCs in the BM. Moreover, we detected a severe reduction in HSPCs in the spleen. It is likely, therefore, that the hypersusceptibility to myelosuppression of LPA_4_-deficient mice was also partly caused by these “basal” impairments of steady-state hematopoiesis in addition to the attenuated emergency hematopoiesis. As far as we know, no gene-targeted knockout mouse lines have been reported to have hematopoietic phenotypes similar to that of LPA_4_-deficient mice, although gene targeting in mice has allowed investigators to reveal hematopoietic functions of various genes.

In relation to the abnormal BM hematopoiesis in LPA_4_-deficient mice, we would like to note that both the ratios of granulocyte and macrophage progenitors to HSPCs and the absolute number of granulocyte/monocytes were mildly increased in the BM of LPA_4_-deficient mice compared with WT mice. In line with the phenotypes, we found that *Spi1* gene (encoding PU.1) was down-regulated significantly in HSPCs of LPA_4_-deficient mice compared with those of WT mice. PU.1 is the transcription factor that suppresses early granulocytic development in adult mice^34^. Together, these results suggest that LPA_4_ deficiency promotes the differentiation of HPCs into granulocyte and macrophage progenitors, also contributing to the reduced numbers of HPCs.

Hematopoiesis during embryonic development occurs in the fetal liver, and then, postnatally, the HSCs in the fetal liver migrate to the BM^35^, where CXCL12 is abundant and acts as an essential factor for HSC retention^36^. Indeed, the *in vivo* deletion of CXCL12 decreased and increased the number of HSCs in the BM and of that in the spleen and PB, respectively^36^. It is worth mentioning that compromised HSC homing was observed in the spleen of naïve LPA_4_-deficient mice, although the BM of these mice contains the normal number of HSCs as well as a normal level of CXCL12 protein. These results suggest that the total absolute number of HSCs in the LPA_4_-deficient fetal liver may be intrinsically reduced, resulting in the compromised extramedullary hematopoiesis observed in the adult. Alternatively, the abnormal angiogenesis during the embryogenesis of LPA_4_-deficient mice^18^ may inhibit the homing of HSCs from the liver to the spleen.

The LPA_4_-deficient stromal cells showed significant decreases in the basal mRNA expression levels of *Scf* and *TN-C in vitro*. These significant decreases were observed even after LPA treatment, although the expression of *TN-C* was comparably induced by LPA treatment in both genotypes, likely via LPA receptor(s) other than LPA_4_. Thus, LPA_4_ signaling in stromal cells may indirectly regulate the intrinsic capacity for the production of SCF and TN-C by affecting unknown processes such as cellular differentiation.

Myelosuppression is a life-threatening adverse event observed during anticancer treatments such as chemotherapy and radiotherapy^37^. Currently, granulocyte colony-stimulating factor is used to promote granulocytic recovery after such anticancer treatments^38^. In the present study, we reveal that LPA_4_ facilitated the regeneration of HSPCs after myelosuppression in mice. It is possible that selective agonism of LPA_4_ could promote multi-lineage hematopoietic recovery and protect cancer patients from myelosuppression.

## Additional Information

**How to cite this article**: Igarashi, H. *et al*. The lysophosphatidic acid receptor LPA_4_ regulates hematopoiesis-supporting activity of bone marrow stromal cells. *Sci. Rep*. **5**, 11410; doi: 10.1038/srep11410 (2015).

## Supplementary Material

Supplementary Information
