# Supplementary material for: The lysophosphatidic acid receptor LPA4 regulates hematopoiesis-supporting activity of bone marrow stromal cells
Source: Sci Rep. 2015 Jun 19;5:11410. doi: 10.1038/srep11410 (PMC4473687; doi:10.1038/srep11410)
Supplement: Supplementary Information [file srep11410-s1.doc]

**Supplementary information**

**The lysophosphatidic acid receptor LPA4 regulates hematopoiesis-supporting activity of bone marrow stromal cells**

Hidemitsu Igarashi, Noriyuki Akahoshi, Takayo Ohto-Nakanishi,

Daisuke Yasuda and Satoshi Ishii*

Author Affiliation

Department of Immunology, Akita University Graduate School of Medicine, Akita, Japan

Author Note

*Correspondence: Department of Immunology, Akita University Graduate School of Medicine, 1-1-1 Hondo, Akita, Akita 010-8543, Japan.

Phone: +81-18-884-6089

Fax: +81-18-884-6444

E-mail: satishii@med.akita-u.ac.jp

**Supplementary Figure 1. LPA receptor mRNA expression in HSPCs and BM stromal cells.** (A-D) Comparison of six *Lpar* mRNA expression in HSPCs (A), PDGFRα^-^Sca-1^-^ (B), PDGFRα^+^Sca-1^-^ (C) and PDGFRα^+^Sca-1^+^ cells (D) by quantitative RT-PCR analysis (*n* = 4-5). ND, not detected.

**
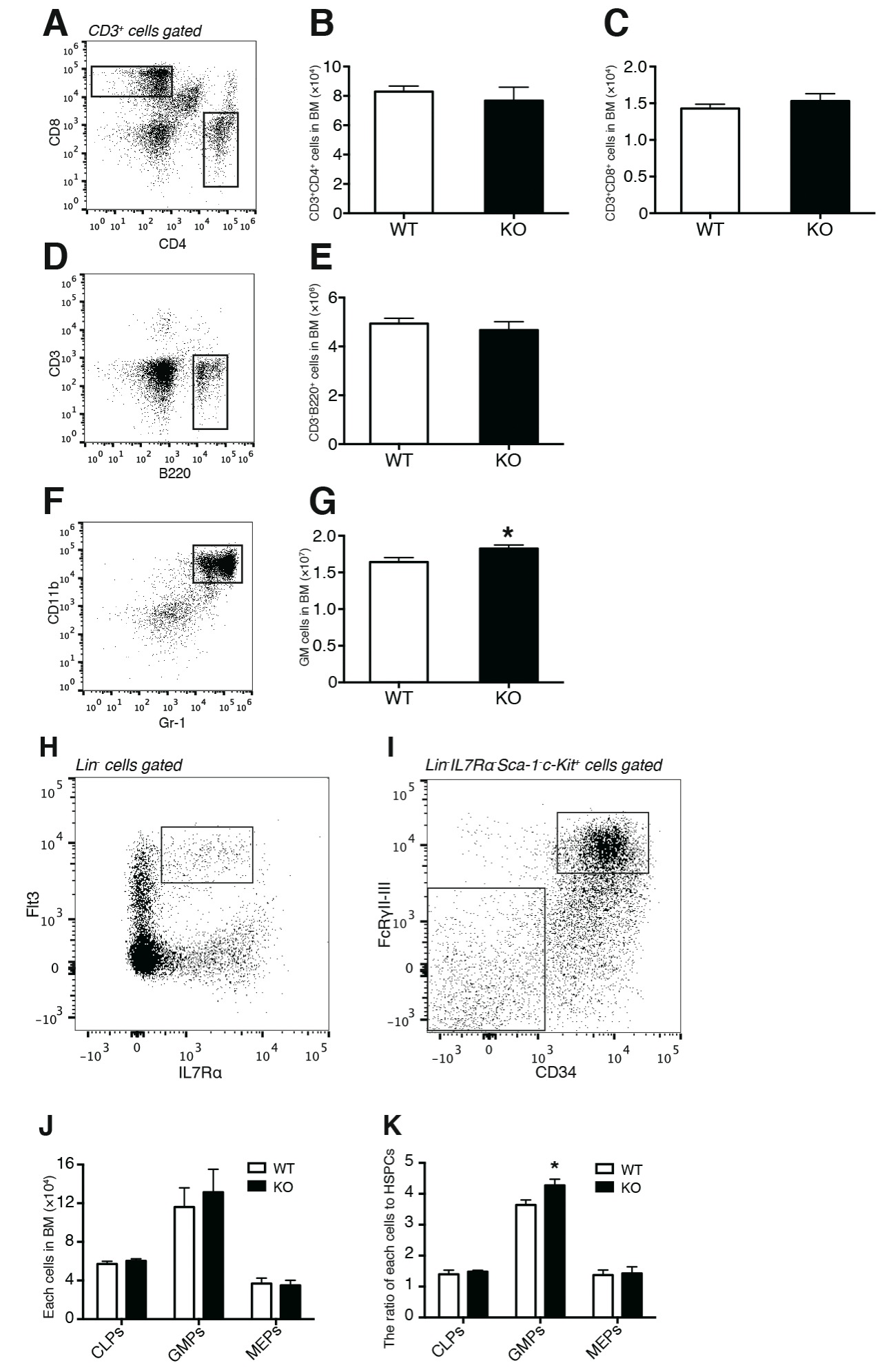
**

**Supplementary Figure 2. Normal steady-state differentiation of HSPCs in LPA4-deficient mice.** (A-G) Representative FACS dot plots and the numbers of CD3^+^CD4^+^ (A and B), CD3^+^CD8^+^ (A and C), CD3-B220^+^ (D and E) and CD11b^+^Gr-1^+^ granulocytes/monocytes (GM cells) (F and G) in the BM of naïve mice (*n* = 8-11). (H and I) Representative FACS dot plots of Lin^-^IL7Rα^+^Flt3^+^ common lymphoid progenitors (CLPs), Lin^-^Sca-1^-^c-Kit^+^CD34^+^FcRγII-III^high^ granulocyte and macrophage progenitors (GMPs) and Lin^-^Sca-1^-^c-Kit^+^CD34^-^FcRγII-III^low^ megakaryocyte and erythrocyte progenitors (MEPs). (J) The absolute numbers of CLPs, GMPs and MEPs (*n* = 8-11) in the BM of naïve mice. (K) The ratios of CLP, GMP and MEP cell numbers to HSPCs (*n* = 8-11). **P* < 0.05.

**Supplementary Figure 3. Colony-forming capacity of the BM cells.** Colony-forming units assay for measuring the capacity of the BM cells from WT and LPA4-deficient mice (*n* = 9). **P* < 0.05.

**Supplementary Figure 4. Comparison of expression levels of various transcripts in HSPCs.** Quantitative RT-PCR for mRNA expression of transcription factor, receptor and cell cycle- and apoptosis-related genes in HSPCs from WT and LPA4-deficient mice (*n* = 5). **P* < 0.05.

**Supplementary Figure 5. Changes in WBC, RBC and PLT counts in LPA4-deficient mice after 5-FU administration.** Kinetic changes in red blood cell (RBC) (A), platelet (PLT) (B) and white blood cell (WBC) counts (C) after 5-FU administration (*n* = 8-11). **P* < 0.05. (Two-way ANOVA followed by Bonferroni’s post-hoc test), ^#^*P* < 0.05. (Interaction, two-way ANOVA).

**Supplementary Figure 6. *Jagged1* mRNA expression level in BM stromal cells.** Quantitative RT-PCR for *Jagged1* mRNA expression in BM stromal cells isolated from WT and LPA4-deficient mice that were treated with or without LPA (*n* = 9-10).

**Supplementary Table 1.** PCR primers used in this study.
